# Identification and characterization of the T cell receptor (TCR) repertoire of the cynomolgus macaque (*Macaca Fascicularis*)

**DOI:** 10.1186/s12864-022-08867-0

**Published:** 2022-09-12

**Authors:** Swati Jaiswal, Sarah K. Nyquist, Shayla Boyce, Tasneem Jivanjee, Samira Ibrahim, Joshua D. Bromley, G. James Gatter, Hannah Gideon, Kush Patel, Sharie Keanne Ganchua, Bonnie Berger, Sarah M. Fortune, JoAnne L. Flynn, Alex K. Shalek, Samuel M. Behar

**Affiliations:** 1 Department of Microbiology and Physiological Systems, Universityof Massachusetts Chan Medical School, Worcester, MA USA; 2 Institute for Medical Engineering & Science, Massachusetts Institute of Technology, Cambridge, MA USA; 3 Ragon Institute of MGH, MIT, and Harvard, Cambridge, MA USA; 4 Broad Institute of MIT and Harvard, Cambridge, MA USA; 5 Computer Science and Artificial Intelligence Laboratory, Massachusetts Institute of Technology, Cambridge, MA USA; 6 Department of Mathematics, Massachusetts Institute of Technology, Cambridge, MA USA; 7 Program in Computational and Systems Biology, Massachusetts Institute of Technology, Cambridge, MA USA; 8 Microbiology Graduate Program, Massachusetts Institute of Technology, Cambridge, MA USA; 9 Department of Microbiology and Molecular Genetics and Center for Vaccine Research, University of Pittsburgh School of Medicine, Pittsburgh, PA USA; 10 Department of Immunology and Infectious Diseases, Harvard T.H. Chan School of Public Health, Boston, MA USA; 11 Ragon Institute of MGH, MIT and Harvard, Boston, MA USA; 12 Koch Institute for Integrative Cancer Research, MIT, Cambridge, MA USA

**Keywords:** T cell receptor, Cynomolgus macaque, Locus map, NHP, Variable region genes

## Abstract

**Background:**

Cynomolgus macaque (*Macaca fascicularis*) is an attractive animal model for the study of human disease and is extensively used in biomedical research. Cynomolgus macaques share behavioral, physiological, and genomic traits with humans and recapitulate human disease manifestations not observed in other animal species. To improve the use of the cynomolgus macaque model to investigate immune responses, we defined and characterized the T cell receptor (TCR) repertoire.

**Result:**

We identified and analyzed the alpha (TRA), beta (TRB), gamma (TRG), and delta (TRD) TCR loci of the cynomolgus macaque. The expressed repertoire was determined using 22 unique lung samples from *Mycobacterium tuberculosis* infected cynomolgus macaques by single cell RNA sequencing. Expressed TCR alpha (TRAV) and beta (TRBV) variable region genes were enriched and identified using gene specific primers, which allowed their functional status to be determined. Analysis of the primers used for cynomolgus macaque TCR variable region gene enrichment showed they could also be used to amplify rhesus macaque (*M. mulatta*) variable region genes.

**Conclusion:**

The genomic organization of the cynomolgus macaque has great similarity with the rhesus macaque and they shared > 90% sequence similarity with the human TCR repertoire. The identification of the TCR repertoire facilitates analysis of T cell immunity in cynomolgus macaques.

**Supplementary Information:**

The online version contains supplementary material available at 10.1186/s12864-022-08867-0.

## Background

Experimental animal models are an essential tool in our pursuit of understanding human physiology. The mouse has been incredibly useful in elucidating the major concepts of immunology, including defining the genetic and molecular basis of immunoglobulin and TCR formation and diversity. As part of this effort, the murine TCR repertoire have been extensively characterized and its knowledge is being used to develop new approaches to facilitate antigen discovery and novel treatments for human disease. However, it is not surprising that many human diseases are inadequately modelled in mice. This has been repeatedly emphasized for cancer and is also true for many infectious diseases. Two important examples are acquired immunodeficiency syndrome (AIDS), which is caused by the Human Immunodeficiency Virus-1 (HIV-1), and COVID-19, which is caused by the SARS-CoV2 coronavirus [1–5]. Mice are naturally resistant to both infections. For HIV research, the field largely turned to nonhuman primates (NHP) as a better alternative because they could be infected with a highly related virus, Simian Immunodeficiency Virus (SIV). Consequently, the rhesus macaque’s TCR locus was among the first NHP TCR locus to be characterized [6]. Cynomolgus macaques have been increasingly used for biomedical research, especially in the fields of neurology, cardiology, and for drug development [7, 8]. Importantly, they are increasingly used for infectious disease research, including as a model for human HIV [9] and SARS-CoV2 infection [5]. Most NHP species, including rhesus macaques, whether in captivity or in the wild, rapidly succumb to *Mycobacterium tuberculosis* infection [10, 11]. However, Flynn’s group finds that following challenge with low dose *M. tuberculosis*, nearly half of infected cynomolgus macaques develop a form of disease that resembles latent TB in people [12–15]. Indeed, the pathology observed among *M. tuberculosis*-infected cynomolgus macaques *recapitulates* the spectrum of human TB pathology [16]. Thus, the cynomolgus macaque is providing insights into human disease not possible with other small animal models.

The tremendous capacity of T cells to recognize diverse antigens has a genetic basis that is inherent in the genomic organization of the T cell receptor (TCR) loci [17]. TCR repertoire diversity arises through genetic mechanisms that minimize the number of genetic elements encoded by the genome while maximizing the potential breadth of expressed TCRs. The germline configuration of TCR genes is not functional. Instead, the TCR loci encode families of variable (V), diversity (D), and joining (J) segments, which undergo rearrangement early during T cell development [17]. Recombination of V, D, and J segments leads to a gene fragment that encodes the V-region domain, which becomes the N-terminus of the TCR protein and determines its antigen specificity. Downstream of the V, D, and J genes are constant (C) region exons, which encode the C-terminus of all TCRs and couples the TCR to the Cluster of differentiation 3 (CD3) protein complex to mediate signal transduction into the T cell. The primary diversity of TCRs arises from the nearly random rearrangement of V, D, and J gene segments, as well as additional diversity that occurs at the V-D and D-J junctions by imprecise recombination and the insertion of non-germline encoded nucleotides (N-regions). TCRs are heterodimers formed by TCRα and TCRβ chains, which are encoded by distinct loci (TRA and TRB, respectively) [18]. The TCRα is encoded when Vα and Jα gene segments recombine; the TCRβ is formed from the recombination of Vβ, D and Jβ gene segments. Additional diversity is created by the random pairing of the TCRα and TCRβ chains. Unlike immunoglobulin genes, somatic mutation does not occur in TCR genes. The potential TCR repertoire varies between animal species and is driven in large part by the number of functional members of V, D, or J segments. In humans, there is the potential to generate 10^15^ unique TCRs.

A second subset of T cells are known as gamma-delta (γδ) T cells, express an alternative TCR, which is encoded by distinct gene segments found in the TRG and TRD loci. The γδ-TCR is structurally similar to the αβ-TCR. Like the TRA and TRB loci, the TRG and TRD loci contain sets of Vγ and Jγ, and Vδ, Dδ and Jδ gene segments, respectively. In general, there are fewer gene segments in the TRG and TRD loci, although the potential diversity is still great because of longer CDR3 regions [19]. γδ T cells remain enigmatic because the antigens they recognize and the antigen presenting molecules that restrict their recognition of antigen are incompletely characterized. Nevertheless, they are identified in the circulation and in the tissues of all mammals, and play important roles in autoimmune disease, and in immunity to infection and cancer [20, 21].

Here we identified the TRA, TRB, TRG and TRD loci of the cynomolgus macaque. Based initially on the homology with human TCR gene segments, and subsequently using the identified gene segments from rhesus macaque and cynomolgus macaque, we systematically identified all the V, D, J, and C gene segments belonging to all four T cell receptor loci. Finally, using the genomic sequences, we designed specific primers for the amplification of the Vα and Vβ regions, and determined which of the V gene segments are expressed in individual subjects. To validate our annotations, we investigated the expressed TCR repertoire in cynomolgus macaques infected with *Mycobacterium tuberculosis*. To minimize the possibility of active infection skewing the TCR repertoire, only samples taken from lung areas where there was no active inflammation or gross infection (i.e., uninvolved lung tissue), were used in the present study. The TCR V-regions used by T cells located in uninvolved regions of lung tissue were analyzed by single cell RNA sequencing. These data will allow the detailed analysis of the T cell responses in cynomolgus macaques as well as comparative immunogenetics studies, comparing different species of macaques, and the evolution of TCR genes among primates.

## Results

### Identification of the Macaca fascicularis (Macfas) TCR loci

**Fig. 1:**
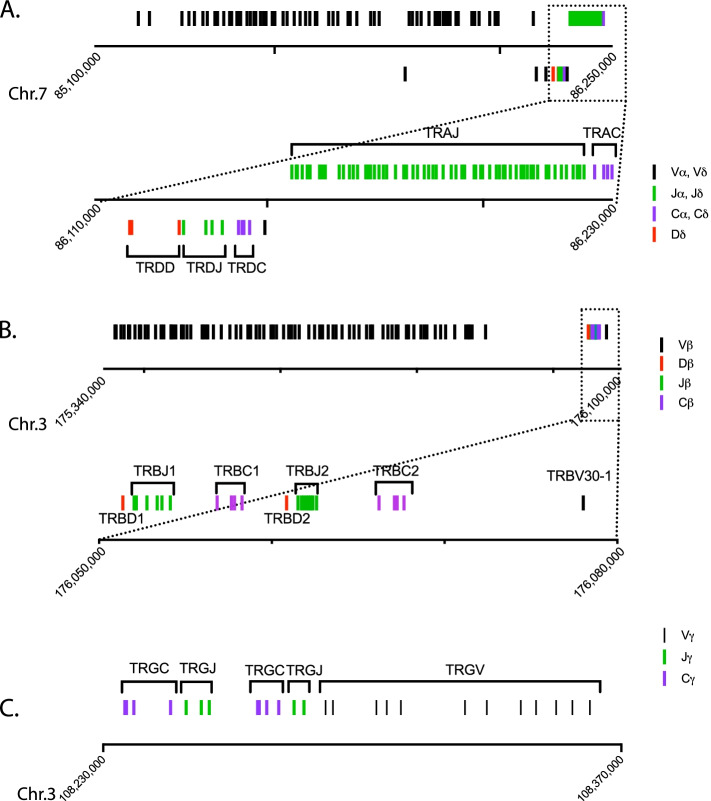
The Macfas TCR loci. Structure of the TCR loci (**A**) TRA/TRD (**B**) TRB and (**C**) TRG loci based on Macaca_fascicularis_5.0. **A** The TRA and TRD loci are interspersed on Chr. 7. The genes above the x-axis belong to the TRA locus; those below the axis belong to the TRD locus. The boxed region is expanded to show greater detail. **B** The TRB locus is located on Chr. 3. The boxed region is expanded to show greater detail. **C** The TRG locus is located on Chr. 3. Each line represents a gene and the distance between them is proportional to their spacing on Chr.7 and Chr.3. The blue boxes represent the 3' region of the AMPH gene and exon 10 of STARD3NL, which are boundaries of the TRG locus. The black lines represent V gene segments; green lines are J gene segments; purple lines represent C region exons, and the red lines represent D gene segments

The Macfas genome assembly Macaca_fascicularis_5.0 (GCF_000364345.1) was used to annotate the different TCR loci. Later, we also used the Assembly MFA1912RKSv2 assembly [22]. Based on nucleic acid sequence homology with the human Cα, Cδ, Cβ, and Cγ gene segments, the TRA and TRD loci were identified on Chr.7, and TRB and TRG loci were identified on Chr.3 (Fig. 1). Subsequently, each human V, D, J, and C gene segment was used to blast the Macfas Chr.7 and 3, to identify homologous gene segments. Similarly, *Macaca mulatta* (Macmul) gene segments were also used to identify homologous genes unique to the macaca genus. Using this approach, we were able to annotate and assemble a map of the Macfas TRA, TRB, TRG, and TRD loci as described in detail below (Fig. 1).

### The Macfas TRA locus

**Fig. 2:**
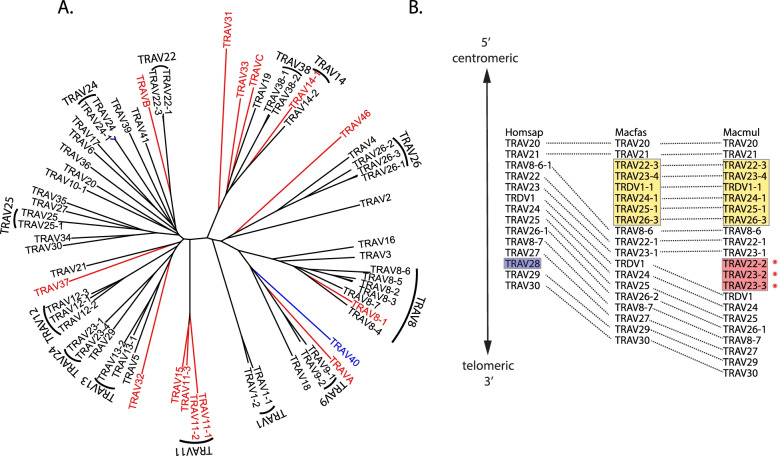
TRAV families. Phylogenetic tree illustrating (**A**) functional genes (black), pseudogenes (red) and pseudogenes in some haplotypes (blue) of the Macfas TRAV locus. The genes clustered together belong to the same family. **B** Comparison of the TRAV/TRDV locus of human, Macfas, and Macmul. The genes that are exclusive to humans are highlighted in purple. Those TRAV genes found in Macfas and Macmul but not in human are in yellow, and the genes are present only in Macmul but absent in Macmul are in red. *, The absence of Macfas orthologs of TRAV22-2, TRAV-23–2, and TRAV23-3, might be a consequence of gaps in the Macfas genome assembly and should not be construed as reflecting evolutionary differences

The structure of the Macfas TRA locus is like the human locus in that it overlaps the TRD locus on Chromosome 7 (Fig. 1A) [23]. We identified 64 TRAV genes in Macfas genome, three more than the 61 human genes but less than the 67 Macmul genes. The two human gene families TRAV7 and TRAV28, each contain a single member and are absent from the Macfas and Macmul TRA locus (Table 1, Fig. 2). Conversely, the TRA loci of Macfas and Macmul have additional genes in the TRAV11, TRAV22, TRAV23, TRAV24, TRAV25, and TRAV26 families. The greater number of Macmul TRAV genes compared to Macfas results from an expansion of the TRAV22 and TRAV23 families (Table 1). Of the 64 Macfas TRAV genes, 15 are pseudogenes and 2 are possible pseudogenes (Table 1, Table S1). There might have been a duplication of a section of the TRA locus. A stretch of six genes (TRAV22, TRAV23, TRDV1, TRAV24, TRAV25, and TRAV26) is repeated, and differentiates the human TRAV locus from the macaque locus (Fig. 2B). The sequences of the affected TRAV genes are not identical, indicating continued evolution over time. It is unknown whether other NHP have such duplications. Second, there are three additional TRAV genes in the Macmul genome assembly that are absent in the Macfas genome. These are Macmul TRAV22-2, TRAV23-2, or TRAV23-3 (Fig. 2B). In searching two different assemblies, we found that six Macmul homologs are missing from the Macfas 5.0 assembly, and four genes are missing from the MFA1912RKSv2 assembly. As both assemblies contain multiple gaps in the TRA/TRD loci, the difference in the number of V-genes in the Macfas and Macmul TRA/TRD locus is likely to be a consequence of limitations in the genome assemblies. A difference in the genomic structure of the Macfas and Macmul TRA/TRD cannot be ruled out but based on the high degree of conservation at the gene level, we believe that such a scenario is unlikely. We identified 61 TRAJ genes, which is the same number as rhesus macaque and human TRAJ genes. There is a high degree of conservation between Macfas and *Homo sapiens* (human) TRAJ gene segments (Table S2). Finally, we compared the TRAC exons from all three species. The Macfas and Macmul TRAC genes have identical amino acid sequences (Figure S1).

**Table 1:**
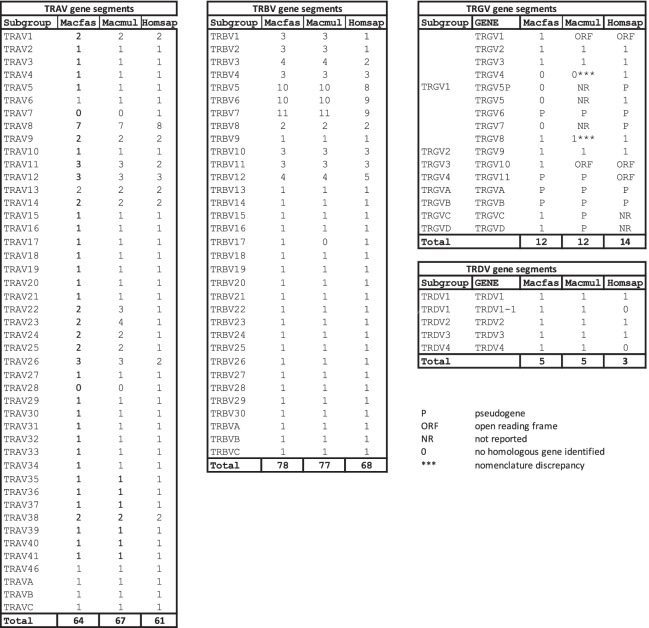
Comparison of Macfas, Macmul and human TRAV, TRBV, TRDV, and TRGV genes

The numerical value for every subgroup represents the number of genes identified

*ORF* Open reading frame, *NR* Not reported, *P* Pseudogene, *F* Functional

*** Nomenclature discrepancy

### The Macfas TRB locus

**Fig. 3:**
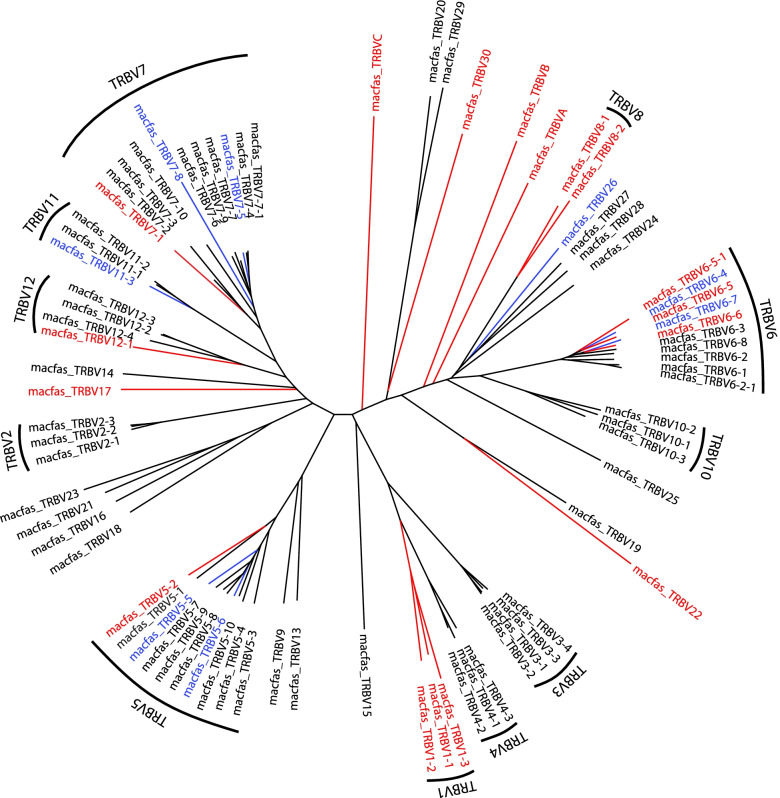
TRBV families. Phylogenetic tree illustrating functional genes (black), pseudogenes (red) and pseudogenes in some haplotypes (blue) of the Macfas TRBV locus. The genes clustered together belongs to the same family

The Macfas TRB locus (Fig. 1B) is similar in structure to the Macmul TRB locus. We identified 78 TRBV genes, compared to 77 annotated Macmul TRBV genes (Table 1 & Table S3). Both are expanded compared to the human species, for which there exists 68 distinct genes. The overall TRBV family structure is similar, with some variation in the number of members and the number of pseudogenes (*n* = 17) and possible pseudogenes (*n* = 8) (Table 1, Fig. 3). The organization of the TRBJ and TRBC genes is similar in all three species, characterized by a duplication of the TRBJ and TRBC genes (Fig. 1B). Comparing the Macfas and Macmul TRBJ gene segments, four (including the TRBJ2.2P ORF) differ by a single nucleotide; the other 10 genes are 100% conserved (Figure S2, Table S4). The TRBD1 and TRBD2 are also 100% conserved between Macfas and Macmul (Table S4). Similarly, there is a high degree of conservation between Macfas and human TRBJ gene segments (Figure S2). Finally, we compared the TRBC exons from all three species. As noted, there are two TRBC genes, TRBC1 and TRBC2, which are 97% identical. The Macfas and Macmul TRBC1 differ by only two bp and the translated sequence is 100% identical; for TRBC2, there is a single amino acid difference (Figure S1).

### The Macfas TRG locus

**Fig. 4:**
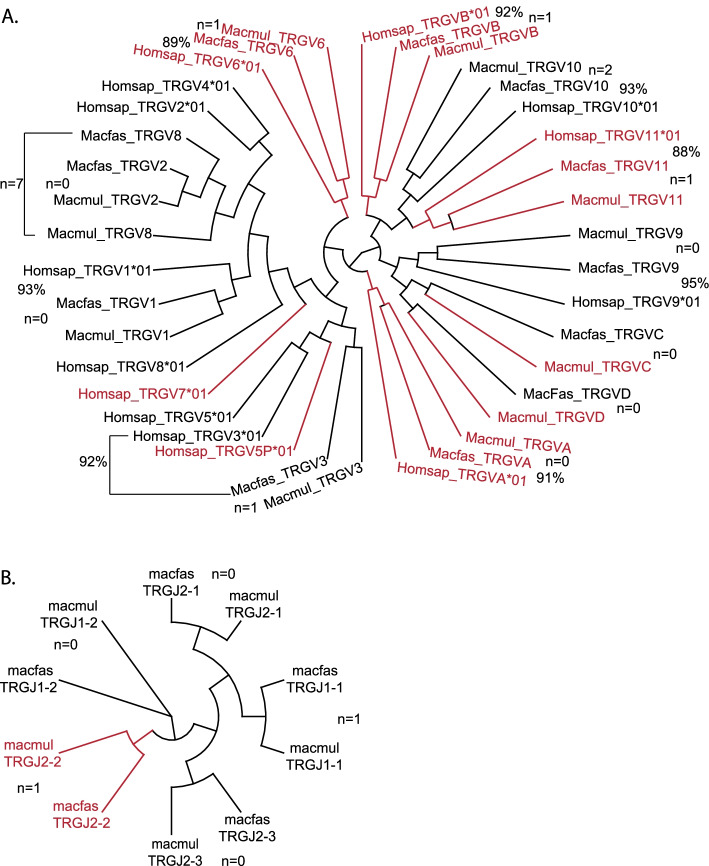
TRGV and TRGJ gene segment homologies. **A** Phylogenetic tree illustrating functional genes (black) and pseudogenes (red) of the Macfas TRGV locus. The number (i.e., “*n* = 1”) is the number of mismatches between the Macfas and Macmul genes. The % is the identity between the Macfas and the human gene. Homologies between other genes of interest are indicated with a dotted line. **B** Phylogenetic tree clustering Macfas and Macmul TRGJ genes

The Macfas TRG locus is located on chromosome 3 (Fig. 1C). We identified 12 TRGV genes of which 6 are predicted to be functional and an additional 4 are pseudogenes (Fig. 4, Table 1, Table S5). These genes were compared to the homologous genes in human and rhesus (Fig. 4). The same 12 genes were found in the Macmul TRG locus. In general, the Macfas and Macmul orthologs had between 0–2 mismatches (i.e., > 99% identity), while the homology between Macfas and human TRGV genes was 88–95%. The two NHP species lacked TRGV4, TRGV5, TRGV5P, and TRGV7, and Macmul had two additional V genes, TRGVC and TRGVD. The human TRG locus has two clusters of J segments and C-region genes [23, 24]; IMGT/LIGM-DB: IMGT000011 (582,960 bp), human (Homo sapiens) TRG locus), and the Macfas and Macmul loci have a similar structure (IMGT/LIGM-DB: IMGT000059 (197,016 bp), rhesus monkey (Macaca mulatta) TRG locus). The five Macfas TRGJ gene segments are very similar to their Macmul counterparts, with between 0–1 bp differences (Fig. 4B). Similarly, there are two Macfas TRGC regions, each encoded by three exons (Table S5). These are highly similar to their Macmul orthologs. Comparing Macfas and Macmul TRGC2 exon 1, 2, and 3, there are 1, 0 and 2 mismatches, respectively, with an overall amino acid sequence identify of 96.5%.

### The Macfas TRD locus

**Fig. 5:**
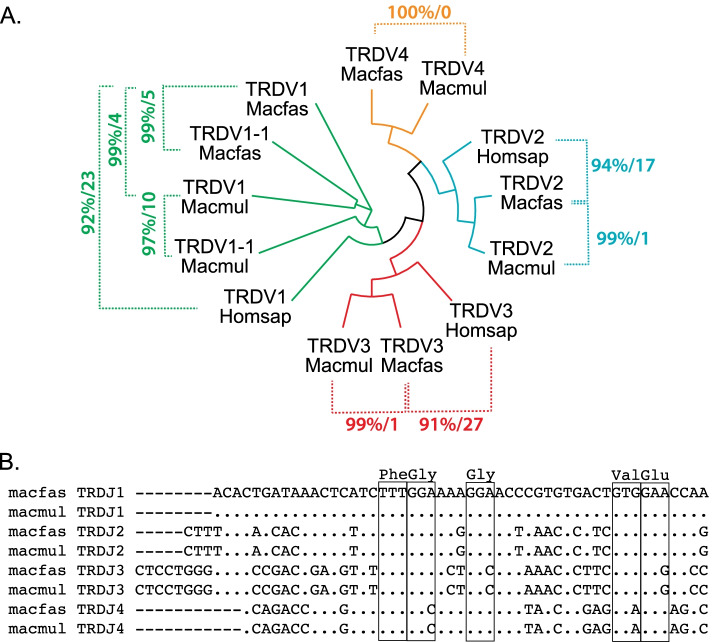
TRDV and TRDJ gene segment homologies. **A** Phylogenetic tree showing the functional genes human, Macfas and Macmul TRDV genes. Comparisons are indicated with dotted lines and the percent identity is indicated followed by the number of sequence mismatches. Each TRDV gene family is color coded. **B** Alignment of Macmul and Macfas TRDJ showing the conserved amino acids (boxed)

The Macfas TRD locus is located on chromosome 7 and overlaps with the TRA locus (Fig. 1A). Three canonical TRDV genes were identified as Macfas homologs of human TRDV1, TRDV2, and TRDV3, with homologies between 91–97% (Fig. 5, Table S6). The two macaque species have an additional gene, TRDV1-1, which is very homologous to TRDV1 (Figs. 2B and 5). We named the Macfas TRDV1-1 based on its orthologous location although its sequence homology is more similar to Macfas TRDV1. A fifth gene, TRDV4, was identified which was 100% homologous to Macmul TRDV4, for which no human ortholog was identified. Three TRDD and four TRDJ Macfas gene segments were identified, as in the human genome (Table S6). These genes are 100% identical to their Macmul homologs (Fig. 5B). Similarly, the single Macfas TRDC region has 100% DNA sequence identity and predicted amino acid sequence as the Macmul TRDC (Figure S1). There is a two amino acid gap, which we suggest is a consequence of the artificial splicing between exons 2 and exon 3.

### The expressed V gene repertoire used by cynomolgus macaque T cells

We determined the expressed TRA and TRB repertoire in cynomolgus macaques infected with *Mycobacterium tuberculosis* by single cell RNA sequencing, The TCR V-regions were amplified using primers as described [25]. Our evaluation of the primers finds that they can be used for analysis of TCRs from rhesus macaque as well (Tables 2 and 3). To determine the functionality of the TRAV and TRBV gene segments we identified, the following criteria were used: (i) Defined L1 exon and L2-V exon, (ii) absence of nonsense or missense mutation, and (iii) encodes a cytosine (C) at position 21–23 followed by tryptophan (W) at position 31–33 of the V exon. The terminal amino acids encoded by a functional TRAV gene is usually CAVR, CAL, or CAF. Similarly, the terminal amino acids encoded by a functional TRBV gene is usually CASSQ, CASSL, or CASSE. Based on these criteria, we initially assigned each V gene to be functional if it met these criteria. If the gene had an internal stop codon, or lacked the conserved C or W residue, it was deemed a pseudogene. Finally, if the gene appeared to be functional, but the L1 or L2 parts of the leader sequence could not be identified, or it lacked consensus splice site for intron A, we designated it an open reading frame (ORF) (Tables S1, S3 and S5).

**Table 2:**
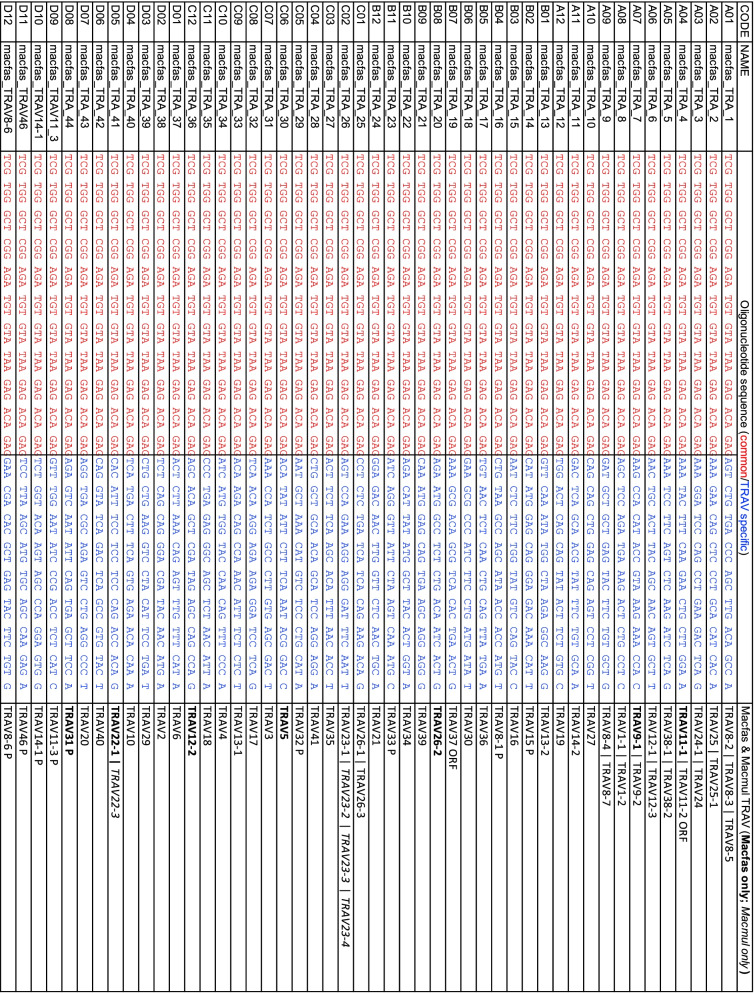
Enrichment primers for TRAV in Macfas and Macmul. The code, name, and sequence of the primers are from [25]. For purposes of this paper, the sequence of each primer is divided into two regions: (i) the 5 ‘handle (in red) which is common to all primers); and (ii) the TRAV-gene specific sequence (in blue). The last column shows the specificity of the primer. Bolded TRAV-genes are specific for macfas; TRAV genes in italics are specific for macmul. P, pseudogene; ORF, open reading frame

**Table 3:**
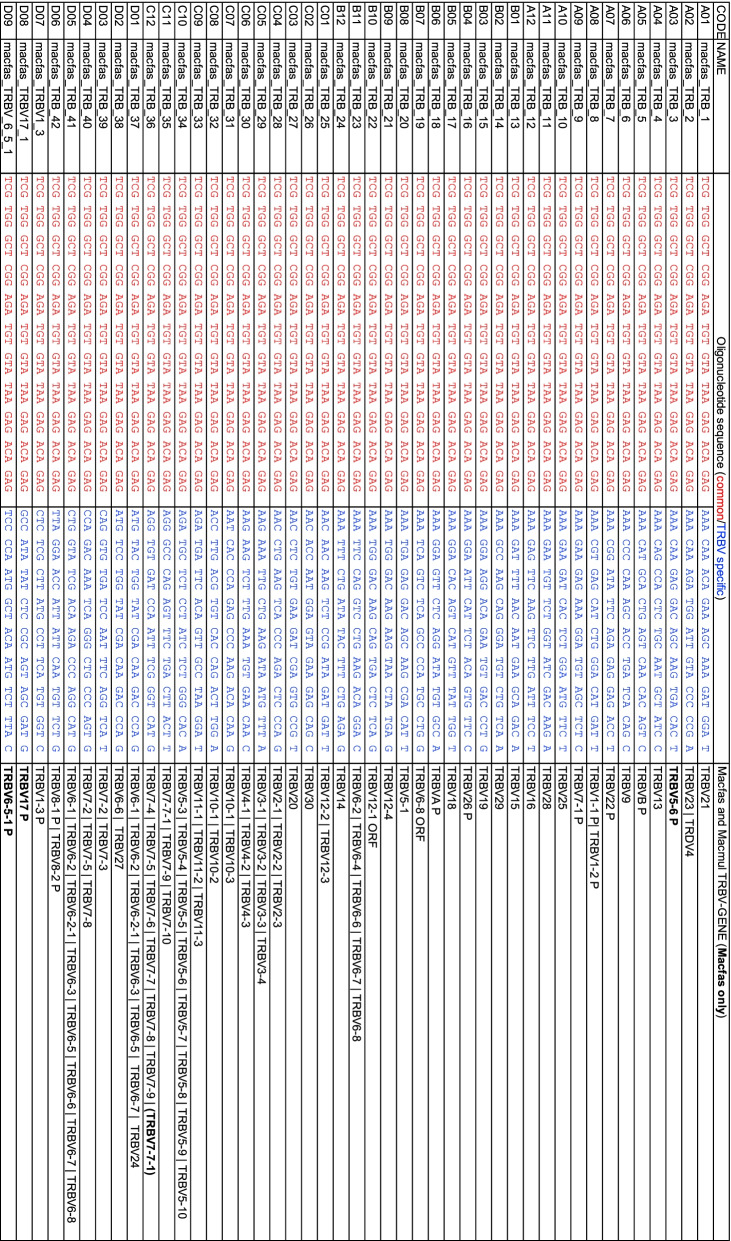
Enrichment primers for TRBV in Macfas and Macmul. The code, name, and sequence of the primers are from [25]. For purposes of this paper, the sequence of each primer is divided into two regions: (i) the 5 ‘ handle (in red) which is common to all primers); and (ii) the TRBV-gene specific sequence (in blue). The last column shows the specificity of the primer. Bolded TRBV-genes are specific for macfas. P, pseudogene; ORF, open reading frame

**Fig. 6:**
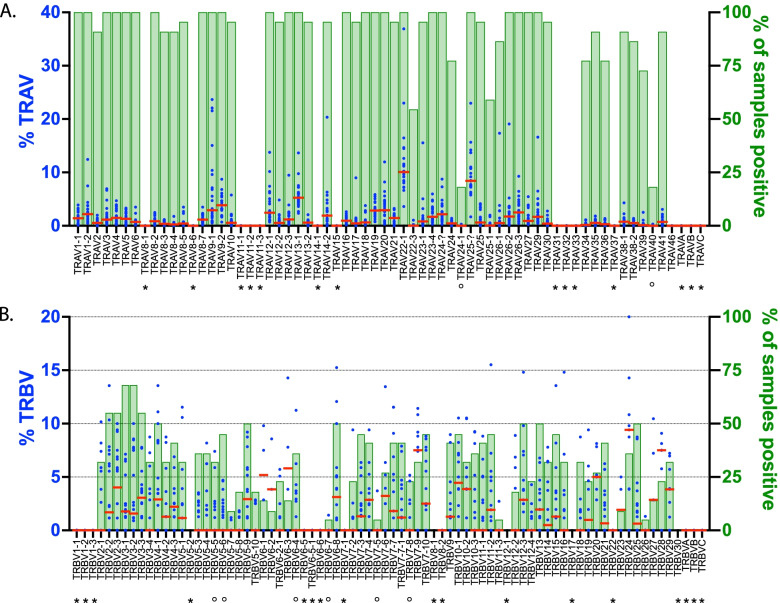
The expressed TRAV and TRBV repertoire. Single cell analysis of lung mononuclear cells from cynomolgus macaques reveals their functionally expressed TRAV and TRBV repertoire. Each dot represents a different subject (*n* = 22). All samples are from uninvolved lung tissue (i.e., uninfected tissue as observed at autopsy). TRAVs were determined for 22 subjects with a median of 968 cells (interquartile range 496–1833). TRBVs were determined for 21 subjects, with a median of 855 cells (interquartile range 375–1587). The distribution of TRAV (**A**) and TRBV (**B**) V regions segments used by the T cells from each individual was calculated. The values within averaged. Red bar, median (left axis). The number of individuals expressing a given TRAV or TRBV gene was also calculated (right axis). *, pseudogene; °, pseudogene in some haplotypes

To determine the expressed TRAV and TRBV repertoire, cells obtained from the uninvolved lung tissue of 22 cynomolgus macaques infected with *Mycobacterium tuberculosis* was analyzed by single cell TCR sequencing*.* The expressed TRAV (Fig. 6A) and TRBV (Fig. 6B) repertoire was determined for each individual macaque. The percentage of individuals that expressed each gene was also calculated. These data allow assignment of each TRAV and TRBV gene as a functional gene or a pseudogene. Overall, there was a good correlation between genes that were predicted to be pseudogenes (based on premature stop codons) and the lack of representation in the transcribed repertoire. However, there were exceptions. For example, TRBV6-4 was predicted to be a pseudogene but was highly represented in the transcribed repertoire. The expected stop codon at position 85 (TAG) was CAG in the transcribed gene, and thus, encoded a functional glutamine (Q). This difference between the germline and the transcribed gene could be the result of a polymorphism or a sequencing error in the genomic reference sequence. Several other genes had similar behavior and were designated as being functional. The status of V genes designated as ORFs, was changed to ‘functional’ if the V gene was transcribed, or to ‘pseudogene’ if it was not. To determine whether the macfas homologs of TRAV22-2, TRAV23-2, and TRAV23-3, which are missing from the genome assemblies, were used by T cells, we included the sequences of the macmul V gene orthologs in the reference database. The algorithm did not assign any TCRs to the missing genes.

## Discussion

The nucleic acid sequence of recombined V, D, and J gene segments encodes the protein structure of the TCR and contains immunological information about T cell responses. The complementarity determining region 3 (CDR3), defined as the V-D-J or V-J recombination site, is unique to each unique T cell clone, sometimes referred to as a clonotype. Analytical approaches are beginning to predict the antigen specificity based on the primary sequence of the TCR. In the absence of the antigen specificity, the TCR sequence can be used as a surrogate of antigen specificity. As T cells undergo clonal expansion after encountering antigens, TCR sequences are being used to track T cells, monitor immune responses, and identify new antigens for human tumors and pathogens [26–28]. Advances in T cell therapy are being driven by our ability to clone and recombinantly express TCRs, as exemplified by adoptive cell therapy (ACT) [29, 30]. Thus, defining the V, D, and J gene segments is an important step in the analysis of T cell immunity.

We identified and annotated the TRA, TRB, TRG, and TRD loci of the cynomolgus macaque. There is generally more than 90% identity between the different V, D, and J gene segments in the human, rhesus and cynomolgus macaque’s TCR repertoire. We also find that there is expansion of TCR beta locus of Macfas and Macmul compared to human. These differences, which are likely to have occurred by gene duplication [31, 32], may have occurred in response to changes in selective pressure during evolution of the TCR loci [33, 34]. As one might expect, the structure of the different TCR loci is highly conserved between rhesus and cynomolgus macaque. The genomic differences we detected (e.g., Fig. 2A) are more likely to be due to ascertainment bias arising from problems with genomic sequencing and assembly, than true evolutionary events. The TCR conservation between cynomolgus and rhesus macaques can be leveraged in the analysis of the expressed TCR repertoire. We used a set of TRA and TRB primers to enrich expressed TCR genes from *M. tuberculosis* infected cynomolgus macaques. Our analysis of these primers shows them to be suitable for enrichment of TRA and TRB genes from rhesus macaques, and therefore this set of primers can be used for both species of macaques [25]. Similarly, nested primers for rhesus TCR enrichment using the 10X Genomics platform can also be used for cynomolgus macaques as the regions to which they anneal are 100% conserved [35, 36].

## Conclusions

We identified and annotated the TRA/D, TRB and TRG loci of the cynomolgus macaque. The TRA and TRB genomic sequences were used to design primers, and as reference sequences, to amplify and identify TCR sequences expressed by single cells from the lungs of cynomolgus macaques. By using these data to analyze the αβTCRs expressed by mature T cells, we were able to discern which V genes were functional based on their RNA expression. This allowed us to refine and validate our predictions based on the genomic sequences. Altogether, these data show the utility of these TCR reference sequences, and we expect that they will be useful for the study of T cell immunity in cynomolgus macaques.

## Methods

### Source of genomic sequence

The genome of the cynomolgus macaque (NCBI: taxid 9541), also known as the crab eating macaque, has been sequenced and we used assembly Macaca_fascicularis_5.0 (GenBank assembly accession: GCA_000364345.1) [37, 38]. The RefSeq numbers for Chromosomes 3 and 7 are NC_052257.1 and NC_052261.1, respectively. Additional gene sequences were obtained from Assembly MFA1912RKSv2 for Macaca fascicularis (crab-eating macaque) (GenBank assembly accession: GCA_012559485.3) [22]. The formal genus and species name is *Macaca fascicularis*, which we abbreviate as Macfas. The rhesus macaque (i.e., *Macaca mulatta*; Macmul) TCR sequences were obtained initially from the literature [39] and later from IMGT (http://www.imgt.org) [40]. The human (i.e., *Homo sapiens*) TCR sequences were obtained from IMGT. In cases where more than one allele was available, the first allele was used for sequence comparisons.

### Annotation and analysis of Macfas TCR repertoire

To identify the location of the Macfas TCR loci, the human TRAC, TRBC, TRGC, and TRDC were blasted against the Macfas genome. Subsequently, all human gene segments were individually blasted against the Macfas genome. As Macfas gene segments were defined, they were also used to look for other homologous genes. At the beginning of this study, the sequences of the Macmul TCBV genes were available and were used to look for homologous genes [39]. The names of the genes were assigned based on the homology with the human genes, and the location in the genome. The leader sequence (L1 & L2), TRV region, D region and J chain were identified for each gene. The annotation was done by following standard IMGT rules (http://www.imgt.org). Clustal Omega was used for multiple sequence alignments (https://www.ebi.ac.uk/Tools/msa/clustalo/) [41] and visualized using Archaeopteryx for Figs. 2, 3, 4 and 5 [42]. Sequences were entered and tracked in Snap Gene (version 5.0).

### Expressed TCR repertoire of cynomolgus macaques

Cells from bronchoalveolar lavage (BAL), single cell suspensions of lung, or lung tissue, were obtained from cynomolgus macaques infected with *Mycobacterium tuberculosis* and single cell RNAseq libraries were created [43]. Primers were synthesized that were specific for the different TRAV, TRBV, TRAC, and TRBC gene segments based on the genomic sequences described herein and used to enrich and amplify the TCR sequences from T cells in scRNA-Seq libraries generated using 3’ barcoded Seq-Well [25, 44]. Primers were not designed for pseudogenes that had internal stop codons, or for some V genes that were not initially identified. The libraries were sequenced and then aligned to the TCR reference sequences. The samples were analyzed for 48 TRAV and 73 TRBV genes. The V region and J region sequences were mapped using BOWTIE 2 as part of the TCRGO algorithm (https://github.com/ShalekLab/tcrgo/tree/master/tcrgo) [25]. Briefly, reads are aligned with the V and J regions in the reference TCR database, containing the sequences annotated in this report (see Results, below). Each read from a Seq-Well library includes nucleic acid tags that identify the cell of origin (cell barcode) and the transcript of origin (unique molecular identifier, UMI). Reads with matching cell barcode and UMI are merged, and a consensus V and J region mapping is determined based on sequence similarity identified among the majority of reads. A consensus CDR3 sequence is identified from reads with shared mappings.

## Supplementary Information

**Additional file 1: Figure S1.** Constant region homology. Alignment of the amino acid sequence of the TCR constant regions, derived from the in silico splicing of the human, Macfas and Macmul TRAC, TRBC, TRGC, and TRDC exons. Dots represent identity. Amino acids are represented by the 1-letter code. X is undetermined.

**Additional file 2: Figure S2.** TRBJ gene segment homology. Alignment of the nucleic acid sequences of the human, Macfas and Macmul TRBJ genes. Dots represent identity.

**Additional file 3: Table S1.** Macfas TRAV genes. The table includes the genomic order of the Macfas TRAV genes, their genomic coordinates, NCBI accession number, the functional status of each TRAV gene, whether the L1, L2, and V regions were identified, and their amino acid and nucleic acid sequences. F, functional; P, pseudogene; ORF, open reading frame; *, expression not analyzed; I, identified; NI, not identified; N/A, not available. Amino acids are represented by the 1-letter code. X is undetermined; dots represent identity; *, stop codon.

**Additional file 4: Table S2.** Macfas TRAJ sequence. The table includes the genomic order of the Macfas TRAJ genes including their genomic coordinates, their NCBI accession number, the % identity to the nearest human homology, their length, and their nucleic acid sequence. The nucleic acid sequence is arranged to show the triplets encoding the conserved amino acid motif “F/W G X G”.

**Additional file 5: Table S3.** Macfas TRBV genes. The table includes the genomic order of the Macfas TRBV genes, their genomic coordinates, NCBI accession number, the functional status of each TRBV gene, whether the L1, L2, and V regions were identified, and their amino acid and nucleic acid sequences. F, functional; P, pseudogene; ORF, open reading frame; *, expression not analyzed; I, identified; NI, not identified; N/A, not available. Amino acids are represented by the 1-letter code. X is undetermined; dots represent identity; *, stop codon. TRBV6-4 is a pseudogene in the genomic sequence but the expressed gene is function.

**Additional file 6: Table S4.** Macfas TRBJ and TRBD sequences. The different tabs of this spreadsheet list the Macfas TRBJ genes including their genomic coordinates, their NCBI accession number, the % identity to the nearest human homology, their length, and their nucleic acid sequence. The nucleic acid sequence is arranged to show the triplets encoding the conserved amino acid motif “F G X G”. The nucleic acid sequence of Macfas TRBD1 and TRBD2 is shown and compared to their orthologs in Macmul and Homsap.

**Additional file 7: Table S5.** Macfas TRGV, TRGJ and TRGC sequences. The different tabs of this spreadsheet list the Macfas TRGV, TRGJ and TRGC genes, their genomic coordinates, NCBI accession number, the functional status of each TRBV gene, the L1, L2, and V region amino acid and nucleic acid sequences. F, functional; P, pseudogene; ORF, open reading frame; N/A, not available. Amino acids are represented by the 1-letter code. X is undetermined; dots represent identity; *, stop codon.

**Additional file 8: Table S6.** Macfas TRDV, TRDJ, TRDD and TRDC sequences. The different tabs of this spreadsheet list the TRDV, TRDJ, TRDD and TRDC genes, their genomic coordinates, NCBI accession number, the functional status of each TRBV gene, the L1, L2, and V region amino acid and nucleic acid sequences. F, functional; P, pseudogene; ORF, open reading frame; N/A, not available. Amino acids are represented by the 1-letter code. X is undetermined; dots represent identity; *, stop codon.

## Data Availability

All data generated or analyzed during this study are included in this published article [and its supplementary information files].

## Abbreviations

ACT: Adoptive cell therapy
AIDS: Acquired immunodeficiency syndrome
C: Constant
CD3: Cluster of differentiation 3
CDR3: Complementary determining region 3
D: Diversity
HIV-1: Human Immunodeficiency Virus-1
J: Joining
γδ: Gamma-delta
Macfas: *Macaca fascicularis*
Macmul: *Macaca mulatta*
*Mtb*: *Mycobacterium tuberculosis*
NHP: Non-human primates
ORF: Open Reading Frame
SIV: Simian Immunodeficiency Virus
TCR: T cell receptor
V: Variable
