# Supplementary material for: Identification and characterization of the T cell receptor (TCR) repertoire of the cynomolgus macaque (Macaca Fascicularis)
Source: BMC Genomics. 2022 Sep 12;23:647. doi: 10.1186/s12864-022-08867-0 (PMC9465142; doi:10.1186/s12864-022-08867-0)
Supplement: Supplementary file 1 — Additional file 1: Figure S1. Constant region homology. Alignment of the amino acid sequence of the TCR constant regions, derived from the in silico splicing of the human, Macfas and Macmul TRAC, TRBC, TRGC, and TRDC exons. Dots represent identity. Amino acids are represented by the 1-letter code. X is undetermined. [file 12864_2022_8867_MOESM1_ESM.pdf]

Figure S1

```
TRAC
macfas 1   XIQNPDPAVYQLRGSKSNDTSVCLFTDFDSVMNVSQSKDSDVHITDKTVLDMRSMDFKSNGAVAWSNKSDFACTSAFKDS
macmul 1   ................................................................................
homsap 1   .............D...S.K..........QT..........Y.................S............AN..NN.

macfas 81  VIPADTFFPGTESVCDANLVEKSFETDMNLNFQNLSVIGFRILLLKVAGFNLLMTLRLWSS
macmul 81  .............................................................
homsap 81  I..E.....SP..S..VK.........T.................................

TRBC1
macfas 1   EDLKKVFPPKVAVFEPSEAEISHTQKATLVCLATGFYPDHVELSWWVNGKEVHSGVSTDPQPLKEQPALEDSRYCLSSRL
macmul 1   ................................................................................
homsap 1   ...N.....E..........................F.................................N.........

macfas 81  RVSATFWHNPRNHFRCQVQFYGLSEDDEWTEDRDKPITQKISAEVWGRADCGFTSVSYQQGVLSATILYEILLGKATLYA
macmul 81  ................................................................................
homsap 81  .......Q.................N....Q..A..V..IV...A...................................

macfas 161 VLVSALMLMAMVKRKDF
macmul 161 .................
homsap 161 ......V..........

TRBC2
macfas 1   EDLKKVFPPKVAVFEPSEAEISHTQKATLVCLATGFYPDHVELSWWVNGKEVHSGVSTDPQPLKEQPALEDSRYCLSSRL
macmul 1   ...................................................................T............
homsap 1   ....N....E............................................................N.........

macfas 81  RVSATFWHNPRNHFRCQVQFYGLSEDDEWTEDRDKPITQKISAEAWGRADCGFTSESYQQGVLSATILYEILLGKATLYA
macmu  81  ................................................................................
homsap 81  .......Q.................N....Q..A..V..IV.......................................

macfas 161 VLVSALVLMAMVKRKDS--
macmul 161 .................--
homsap 161 .................RG

TRGC2
Macfas 1   DKHLDADVSPKPTIFLPSIAETNLHKAGTYLCLLEKFFPDVIEIHWQEKNSNKVLKSQEGNTMKTNNTYMKFSWLTVPEK
Macmul 1   ................................................................................
Homsap 1   ..Q...................K.Q...............I.K......K..TI.G..........D............E

Macfas 81  SLDKEHRCIVRHENNRNGVDQEIIFPPIKT----------------DVTTVDPKDSFSKDANDALLLQLTNTSAYYMYLL
Macmul 81  ..............................----------------.D.......NY.E.....Q...............
Homsap 81  ...............K..I...........DVTTVDPKYNYSKDAN..I.M....NW......T................

Macfas 145 LLLKSEVYFAIIAVCLLRRTAVCCNGERS
Macmul 145 ..V..........................
Homsap 161 .....V......TC...G...F.....K.

TRDC
Macfas 1   -RQPHTKPSVFVMKNGTNVACLVKDFYPKDIRINLESSKKITEFDPAIVVSPSGKYNAVKLGQYADSNSVTCSVQHNKEV
Macmul 1   X...............................................................................
Homsap 1   XS......................E..........V.............I............K.E...........DNKT

Macfas 80  VYSTDFEVKTNSTDHLKPTETENTKQPSKSCHEPKAIVHAEKVNMMSLTVLGLRMLFAKSVAINFLLTAKLLFL*
Macmul 81  ...................................--......................................
Homsap 81  .H........D....V..K.............K......T...................T..V........F...
```
