# Supplementary material for: Identification and characterization of the T cell receptor (TCR) repertoire of the cynomolgus macaque (Macaca Fascicularis)
Source: BMC Genomics. 2022 Sep 12;23:647. doi: 10.1186/s12864-022-08867-0 (PMC9465142; doi:10.1186/s12864-022-08867-0)
Supplement: Supplementary file 2 — Additional file 2: Figure S2. TRBJ gene segment homology. Alignment of the nucleic acid sequences of the human, Macfas and Macmul TRBJ genes. Dots represent identity. [file 12864_2022_8867_MOESM2_ESM.pdf]

TRBJ1

```
1.1     macfas TGAACACTGAAGCTTTCTTTGGACAAGGCACCAGACTCACAGTTGTAG
        macmul ............................................T...
        homsap ................................................

1.2     macfas CTAACTATGACTACACCTTCGGTTCAGGGACCAAGTTAACTGTTGTAG
        macmul ................................................
        homsap .........G...............G.......G......C.......

1.3     macfas TTCTGGAAACACCGTGTATTTTGGAGAGGGAAGTCGGCTCACTGTTGTAG
        macmul ..................................................
        homsap C............A.A..................T...............

1.4     macfas CAACTAATGAAAAACTGTTTTTTGGCAGTGGAACCCAGCTCTCTGTCTTGG
        macmul ...................................................
        homsap ...................................................

1.5     macfas TAGCAATCAGCCCCAGTATTTTGGAGATGGCACTCGACTCTCCGTCCTAG
        macmul ..................................................
        homsap ................C.......T.....G............A......

1.6     macfas CTCCTATAATTCGCCCCTCCACTTTGGGAACGGGACCAGGCTCACTGTGACAG
        macmul ...................................................G.
        homsap ............A.................T......................
```

TRBJ2

```
2.1     macfas CTCCTACAATGAGCAGTTCTTTGGGCCAGGCACACGGCTCACCGTGCTAG
        macmul ..................................................
        homsap .....................C........G...................

2.2     macfas CCAACACCGCGCAGCTGTTCTTTGGAGAAGGCTCTAGGCTGACCGTGCTGG
        macmul .T.................................................
        homsap .G.......G.G.......T..........................A....

2.2P    macfas CTGAGAGGCGCTGCTGGGCGTCTGGGCCGAGGACTCCTGGTTCTGG
        macmul .................................G............
        homsap ...........................G..................

2.3     macfas AGCACAGATCCGCAGTATTTTGGCCCAGGCACCCGGCTGACAGTGCTCG
        macmul .................................................
        homsap .........A.......................................

2.4     macfas AGCC-AAAACACTCAGTACTTCGGCGCCGGGACCCGGCTCTCAGTGCTGG
        macmul ....-.............................................
        homsap ....A......T......................................

2.5     macfas ACCAAGAGACCCAGTACTTCGGACCAGGCACGCGGCTCCTGGTGCTCG
        macmul ................................................
        homsap ......................G.........................

2.6     macfas CTCTGGGGCCAGCGTCCTGACTTTCGGGGCCGGCAGCCGGCTGACCGTGCTGG
        macmul .....................................................
        homsap ...........A.........................A...............

2.7     macfas CTCCTACGAGCAGTACTTCGGGCCGGGCACCAGGCTCACAGTCATAG
        macmul ...............................................
        homsap .......................................G....C..
```

Supplemental Figure S2
